# Electrophysiological Evidence for A Number–Action Mapping in Infancy

**DOI:** 10.3390/brainsci12111480

**Published:** 2022-10-31

**Authors:** Gisella Decarli, Pia Rämä, Lionel Granjon, Ludovica Veggiotti, Maria Dolores de Hevia

**Affiliations:** Integrative Neuroscience and Cognition Center, Université Paris Cité, CNRS, F-75006 Paris, France

**Keywords:** numerosity, action, grasping, EEG, ERP, infancy, cueing paradigm

## Abstract

In the last decades, a growing body of literature has focused on the link between number and action. Many studies conducted on adult participants have provided evidence for a bidirectional influence between numerosity processing and grasping or reaching actions. However, it is not yet clear whether this link is functional in early infancy. Here, we used the event-related potential (ERP) technique to record electrical activity of the brain in response to number–hand pairings. We implemented a cueing paradigm where 3- to 4-month-old infants observed images showing either congruency (e.g., a large numerosity primed by a large hand opening) or incongruency (e.g., a large numerosity primed by a small hand opening). Infants’ brain activity was modulated by the congruency of the pairings: amplitudes recorded over frontal and parietal-occipital scalp positions differed for congruent versus incongruent pairings. These findings suggest that the association between number and hand action processing is already functional early in life.

## 1. Introduction

At birth, humans possess the ability to represent large quantities in an approximate way [1,2]. This ability develops in precision until adulthood following specific ratios: newborns can differentiate numerosities differing by a ratio of 1:3, 6-month-old infants by a 1:2 ratio, 9-month-olds by a 2:3 ratio, and adults are able to tell apart 9 vs. 10 [3,4,5]. In the last decades, this number sense, which is supported by the approximate number system (ANS), has received increasing attention due to its hypothesized crucial role in the acquisition of early mathematical skills ([6] for a review) and in the emergence of a specific learning disability in the math domain (i.e., developmental dyscalculia; [7]).

The ANS has also been connected to other cognitive abilities. Originally, Gallistel [8] theorized a common metric used for both discrete (i.e., numbers) and continuous dimensions (i.e., space and time), allowing various species to execute arithmetic operations. In this view, a common currency would account for the connection between different mental magnitudes. Evidence for this theory has been provided by studies implemented in animals, e.g., in the common magnitude of space and time in pigeons [9] or in the link between discrete and continuous magnitudes (number and size) in honeybees [10]. 

In the same vein, Walsh and colleagues proposed the ATOM view (a Theory of Magnitude), i.e., a common magnitude metric underlying representations of number, space, and time, which are used to correctly plan and implement actions [11,12,13]. According to the ATOM view, these quantitative dimensions need to interact with each other to finalize an action, and the common magnitude area should be localized in the parietal cortex. More recently, other authors have proposed a similar perspective [14]. According to them, in the numerosity domain, the action and perception systems share common neural mechanisms: they postulate the existence of a common system that processes numerosities, which can be generated internally (by motor routine actions) and externally (by other sources, such as visual and auditory ones). This theory finds its roots in evidence regarding humans’ and non-humans’ ability to represent the numerosity of internally generated events [15,16], the common neural path for both processes (in the parietal cortex; [17,18]), and the phenomenon of psychophysical adaptation in a sensorimotor numerosity mechanism [19,20].

In line with the ATOM view as well as the theory proposed by Anobile and colleagues, a large literature has provided evidence for a link between number and space (see for a meta-analysis, [21]), that is functional from birth [22,23,24,25]. Numerosities can be connected to spatial representations through two mappings, one where numbers are associated to representations of spatial extent and another to lateralized spatial positions [26]. Human newborns and infants are able to map numerical representations into spatial lengths, generalizing a positive relation of these two dimensions from exemplars to new values and showing a spontaneous preference for congruency between numbers and lengths [24,27]. On the other hand, the ability to map numerosities into lateralized spatial representations has been demonstrated in both infants and in newborns, and even in non-human species [28,29]. 

In this study, we specifically aimed to test the link between number and hand action in infancy. At the behavioral level, evidence for bidirectional interference has been provided in a series of studies in adults. Some authors have shown that when participants were asked to perform a grip aperture, the numerosity presented just before the action influenced the motor output, i.e., a hand closure was started faster in response to small numerosities [30,31,32]. In the same vein, the presentation of different numerosities affected the estimation of rod grasping according to the numbers’ magnitude [33]. The influence of action observation on numerical processing has also been reported. Some authors have demonstrated that observing a grip closing or a grip aperture influences the magnitude of the numbers produced by participants in a number-generation task [34]. Similar results were found when participants had to name one of two Arabic digits (one odd and one even) after observing hand movements, mimicking either opening or closing grips. Participants responded more slowly to the large numbers than to the small ones when they were preceded by a grip closing [35]. More recently, Ranzini and colleagues [36] found slower reaction times (RTs) in a digit comparison task (i.e., indicating whether a digit is smaller or larger than five) after executing a pointing movement. Therefore, all these studies indicated a bidirectional link between perception/execution of motor actions and numerical magnitudes.

At the neural level, studies have separately tested the neural substrates of numerosity perception and hand actions, demonstrating the role of parietal cortex in processing both sources of information [37,38,39]. Interestingly, one study has investigated both mechanisms to investigate the possible activation of overlapping cortical areas. Monkeys were trained to perform cyclical movements (i.e., five times) and cellular activity was found in the superior parietal lobule [40], demonstrating that this area is activated when performing numerically-based behavioral tasks. Therefore, across species, we can find the involvement of the parietal cortex in numerosity and action processing. 

In humans, some authors have found similar cerebral activations for numbers and hand actions [41,42]. A recent meta-analysis showed that neural activations for symbolic number processing and hand action shared four frontoparietal areas, two in the left and right parietal regions and two in the frontal regions [43]. This common network would presumably extend to non-symbolic numbers as well [44]. Neighboring areas in the parietal cortex are engaged in non-symbolic numerical processing, as well as in visuo-motor tasks, which are already active in infancy/childhood [45,46,47,48]. 

Evidence about a shared mechanism for both numerical and action processing in developmental populations is, however, very limited. Some studies have provided evidence that infants are able to successfully process the information about objects’ size in order to perform manual actions [49,50,51]. Interestingly, and in line with these findings, before the first year of life infants can anticipate the goal in an observed action setting by using the information of the amplitude of the hand opening [52]. Moreover, a line of research has demonstrated the distinction between dorsal and ventral processing. One previous study provided behavioral evidence supporting the functional distinction between ventral versus dorsal pathways. In particular, two distinct cognitive paths have been suggested to be at play when performing a variety of tasks, whereby the ventral stream is thought to be recruited in object identification processing, while the dorsal stream is thought to be involved in both numerosity comparison and finger movements [53]. Interestingly, behavioral evidence for a distinction between these two pathways has been demonstrated in 4-month-old infants, as they are able to retain information provided by either the dorsal or the ventral streams, but fail to retain information provided contemporarily by both streams (i.e., objects’ location vs. surface features, respectively; [54]). Further evidence for this differentiation comes from electroencephalogram (EEG) study in infancy. Izard and colleagues [47] found distinct neural responses for number vs. object changes, and using cortical source reconstruction, they found the former involving parietofrontal network and the latter involving ventral temporal areas.

Recently, one study has assessed for the first time the link between number and action in infancy [55]. Seven- to nine-month-old infants were habituated to either congruent (e.g., large numerosities paired with large hand openings) or incongruent (e.g., large numerosities paired with small hand openings) number–hand pairings. In the test trials, participants observed both types of pairings, congruent and incongruent, constructed from new examples of numerosities and hand openings. Results showed that only infants habituated to congruent number–hand pairings looked significantly longer at the test trial showing an incongruent pairing, suggesting they abstracted the congruent link and applied it to new instances. Conversely, when exposed to an incongruent pairing during habituation, infants did not show evidence of learning nor generalizing the link to new stimuli. These findings provide initial evidence for infants’ spontaneous ability to link the dimensions of number and action and to use this knowledge in a productive way, provided that magnitude changes across the two dimensions varied in a positive, congruent way [55].

Here, we used the event-related potential (ERP) technique to record electrical activity of the brain in response to congruent and incongruent number–hand pairs in 3- to 4-month-olds. The ERPs provide a temporally accurate measure of brain activity underlying cognitive processes. The technique is especially suitable to investigate infant cognition, as it allows us to study cognitive processes without overt motor responses. In line with recent studies in infants [56,57], we implemented a cueing paradigm, where infants were presented with images displaying congruent (e.g., a large numerosity primed by a large hand opening) and incongruent (e.g., a large numerosity primed by a small hand opening) number–hand couplings. The rationale behind these stimuli choices was to convey information of relative magnitude, as opposed to conveying information of an action itself, by varying the amplitude of a hand opening. These stimuli were largely based on stimuli used in the past with adults (see e.g., [35]). By showing infants static images of hand shapes that convey information of relative magnitude (i.e., large opening amplitude vs. small opening amplitude) immediately followed by relative numerical magnitude (i.e., large vs. small numerosity), we tested whether infants spontaneously associate and integrate the two sources of magnitude information. The choice of using simple openings of the hand without showing goal-directed actions was motivated by the fact that human infants (probably starting in the womb) are able to produce this simple action of hand opening very early in life, and even adapt it to the objects’ featural properties ([58,59]). Moreover, we presented the hand shape and the numerical array sequentially, which contributed as well to deter the infant from interpreting the stimuli as being part of a goal-directed action setting, therefore inviting infants to create abstract magnitude representations based on the opening of the hand and the quantities represented by the numerical arrays. 

Regarding the EEG activity, there are a number of ERP components that have been associated with violation of expectations or learned associations in meaningful contexts. The N400 component has been traditionally associated with processing of meaning in various linguistic and non-linguistic contexts (see for a review, [60]). The N400 effect is described as a greater negativity in response to unexpected than to expected events, and it typically occurs around 200–600 ms after stimulus onset over the central-parietal scalp positions. Recent evidence shows that the N400 is functional already in early childhood. In the language domain, the N400 effect has been found in infants and toddlers using a picture-word matching task, where spoken words were associated with congruent or incongruent pictures (e.g., [61,62]). Moreover, this component has been observed in auditory semantic priming tasks (e.g., [63,64,65]), where toddlers were presented with semantically related and unrelated word pairs. The earliest age at which an N400 effect has been found in a lexical-semantic paradigm is at 6 months [61]. Similar differences in brain signals between expected and unexpected events have been reported in visual action perception studies with infants. For example, observation of sequences of actions that could be either expected or not (e.g., simulation of eating with a spoon that is directed either towards the mouth or the forehead), revealed differential ERPs in response to anticipated versus unanticipated final images by 9 months of age [66]. In another study, a violation of a previously learned association elicited an enhanced Negative Central (Nc) ERP component over the frontal and central recording sites in response to deviant action pairs in 8- to 11-month-olds, while no evidence for posteriorly distributed N400 was observed [67]. Earlier research suggests that the magnitude of the Nc is modulated by attentional engagement, stimulus novelty, or contextual change ([68]; see also [69]). Finally, another component that has been associated with processing of expected vs. unexpected outcomes is the positive slow wave (PSW). In a recent study, a more pronounced PSW component over the frontal and the central recording sites was found in response to unexpected, compared to expected, action outcomes in 5-month-olds [70]. In some studies, this component has been linked to the perception of familiar vs. unfamiliar stimuli and interpreted as an enhanced activity involved in the encoding of unfamiliar outcomes. 

Based on previous literature, we formulated the following hypotheses. If infants at this early age are already able to integrate the two sources of magnitude information conveyed by the opening amplitude of a hand shape, and by the numerical array, and to create expectations on how they are related (i.e., expectation of congruency), then we should observe different ERP amplitudes when infants are observing congruent versus incongruent hand–number pairings. This finding would suggest that an integration between these two types of magnitudes at an abstract or conceptual level is already functional at the early stages of development. On the other hand, if infants are not yet able to create expectations of congruency between the two magnitudes conveyed by the hand amplitude and by the numerosity, then we should observe similar electrophysiological signals across congruent and incongruent pairings.

We expected to observe a difference in the amplitudes of ERPs in response to congruent and incongruent pairings, suggesting that an integration between these two types of information is already functional in the early stages of development. 

## 2. Materials and Methods

### 2.1. Participants

Thirty-three healthy full-term 3- to 4-month-old infants (15 girls and 18 boys, mean age = 3 months 24 days, range = 2 months and 27 days–4 months and 4 days) participated in the study. Data from fourteen infants were rejected due to fussiness and/or noisy electroencephalogram (EEG) recording (less than ten accepted trials in one experimental condition). A final sample of 19 infants were considered for the analyses (8 females and 11 males, mean age = 3 months 28 days, range = 3 months–4 months and 4 days). The mean number of years for maternal education was 15.6 (range from 9 to 19 years). 

Participants were recruited by letters that were sent based on birth records provided by the municipality of the Paris city. Parents gave a written consent for participating in the study. The protocol was carried out in accordance with the ethical standards of the Declaration of Helsinki and approved by the Université Paris Cité Ethics Committee.

We tested 3- to 4-month-old infants, an age when infants do not benefit from extensive experience in performing actions in an accurate and efficient way. However, despite the immature state of action-related abilities at this age, infants are not entirely naive about the basics of actions. Human newborns possess a very roughly ability to perceive/interpret a hand movement action, as they display higher looking times at a hand performing a movement away from the body and towards the object to be grasped [71]. In our study, we did not depict a goal-directed action but instead presented static, simple openings of a hand, or hand shapes, displaying two different opening amplitudes; infants from early in life execute openings of the hand and, in some cases, are even able to adapt them, albeit not accurately, to objects’ featural properties [58,59].

### 2.2. Stimuli

**Figure 1:**
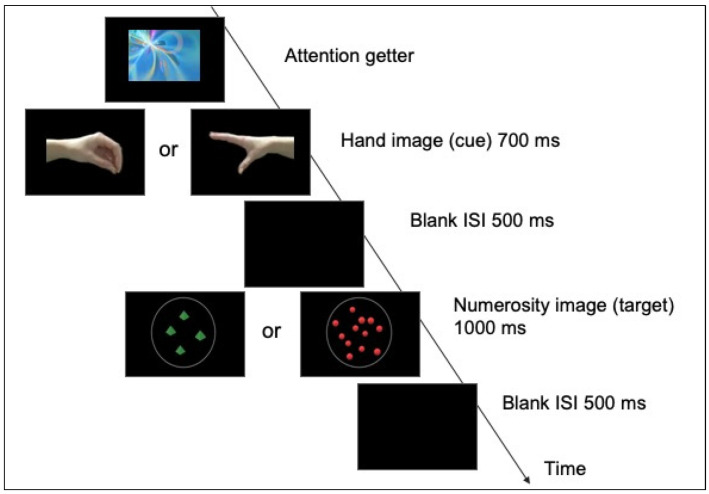
Schema of trial procedure: after the presentation of an attention getter, the hand image (cue) that depicted either an open or a close grip, oriented towards the left or right, was followed by a non-symbolic numerosity array containing either 4 or 12 elements. Congruent pairings were those in which an open grip was followed by an array of 12 and a close grip by an array of 4; the reverse association was considered an incongruent pairing.

Each trial was composed by the sequential presentation of two images, one displaying a hand and one a numerosity (see Figure 1).

The hand image (cue) could vary on the magnitude of the opening (closed vs. open), with different spatial orientations (right vs. left). Different hand models were used (lateral and frontal views of a hand). The width of the hands could vary between 14.3° and 17.4° visual angle, while the height remained roughly constant around 6.1° visual angle. The numerosity image (target) consisted of visual arrays of either 4 or 12 elements, which varied in shape and color (red dots, blue cubes and green pyramids). Half of the target images were controlled for individual size and the other half for the total surface area. In the area constant condition (i.e., area remains constant for all the images), the diameters of 12 items varied between 1.37° and 1.72° visual angle, while the diameters of 4 items could vary between 2.34° and 2.91° visual angle. In the size constant condition the diameters could vary between 1.64° and 2.05° visual angle. We included different sets of numerical displays so that infants would refrain from basing their magnitude judgements on non-numerical features of the visual array (such as dots’ size or total surface area), as well as to increase the variability (and interest) of the stimulation.

### 2.3. Procedure

Infants were tested in a dimly illuminated room. During the experiment, infants were positioned on a parent’s lap at approximately 70 cm from the monitor (resolution: 1920 × 1080). A camera (Logitech HD 720p) placed above the monitor was used to show the image of the baby to a pc outside the room, allowing the experimenter to check the infant’s attention. The stimuli were presented and controlled by a computer with E-Prime 2.0 (Psychology Software Tools, Inc. Pittsburgh, PA, USA).

Each trial started with a visual and colorful “attention-getter”, displayed in the center of the screen and randomly selected among different animations. As soon as the infant oriented the attention towards it, the experimenter pressed a key to start the trial. The attention-getter was replaced by the image of a hand, followed by an image of a numerosity. The hand image (cue) was presented for 700 ms, followed by the target image (numerosity) that remained on the screen for 1000 ms. The interstimulus interval (a black screen) was 500 ms. The target image (numerosity array) could be either congruent (i.e., open hand followed by an array of 12 elements) or incongruent (i.e., open hand followed by an array of 4 elements) with the cue image (hand). Congruent and incongruent trials were randomly presented. In order to obtain as many trials as possible from each infant, there was no restriction in the number of blocks or trials shown: they were played as long as the infant was attentive.

### 2.4. EEG recording and Pre-Processing 

Continuous electroencephalogram (EEG) was recorded (band-pass was 0.1–100 Hz, sampling rate was 250 Hz) from 128 electrodes using a Geodesic Sensor Net (GSN, NetStation EGIS V2.0, Magstim EGI, Eugene (OR), USA). Impedances were kept below 50 kΩ. EEG was bandpass filtered (0.3–30 Hz) and segmented (1000 ms, beginning 100 ms before the target image). The 100-ms pre-stimulus period determined the baseline for amplitude measures. Ocular artifacts were removed with an ocular artifact removal (OAR) algorithm [72]. The epochs including artifacts (eye-movements, blinks, motion artifacts exceeding ± 200 μV in any channel) were excluded. The epochs were averaged separately for each subject and type of trial (congruent and incongruent) pairing. The averaged waveforms were re-referenced to the average reference and baseline corrected. The mean number of accepted trials was not significantly different among the two conditions (one-way ANOVA: *F*(1,18) = 2.35, *p* = 0.14; congruent trials: *M* = 21.21, *SD* = 7.73; incongruent trials: *M* = 19.89, *SD* = 6.71). Overall, the mean number of accepted trials per subject was 20.55 (range: 10–37). 

### 2.5. Data and Statistical Analyses

Similarly to earlier studies (e.g., [73]), we extracted amplitudes from two time windows: 200 ms to 500 ms (early window) and 500 ms to 1000 ms (late window). A repeated-measures analysis of variance (ANOVA) included Trial Type (congruent/incongruent numerosity-hand’s opening pairing), Recording Area (frontal/parietal), and Laterality (left/midline/right) as within-subject variables. Based on previous studies [63,66,67], the mean amplitudes extracted from 42 electrodes over the frontal and the parietal-occipital recording areas were included in the statistical analyses. 

The frontal and the parietal-occipital recording sites included the following electrode positions: 20, 24 (F3), 28 (FC5), 29 (FC3), 35, 36 (C3) and 30 (C1) (frontal left); 12, 5, 6 (FCZ), 13 (FC1), 112 (FC2), 7 and 106 (frontal midline); 104, 105 (C2), 110, 111 (FC4), 117 (FC6), 118 and 124 (F4) (frontal right); 59, 60 (P1), 66, 70 (O1), 65 (PO7), 69 and 73 (parietal-occipital left); 72 (POZ), 62 (PZ), 75 (OZ), 71, 74, 76, 82 (parietal-occipital midline); 85 (P2), 84, 83 (O2), 91, 90 (PO8), 89 and 88 (parietal-occipital right). The statistical analyses were conducted with R [74]. The Greenhouse-Geisser correction was applied for non-sphericity when appropriate. 

## 3. Results

**Figure 2:**
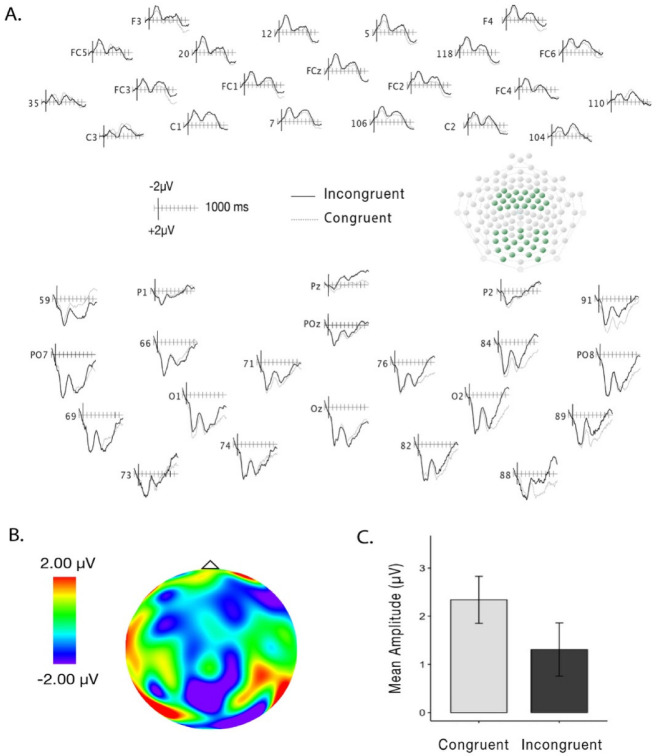
(**A**) Grand-averaged waveforms for congruent (dashed lines) and incongruent trials (solid lines) over the frontal and parietal-occipital areas over the late time-window. (**B**) Difference wave (incongruent-congruent) topographical map displaying activity over the late time-window. (**C**) Mean amplitudes across frontal and parietal-occipital areas for congruent and incongruent trials in the late time-window. Error bars represent standard error from the mean.

There was no main effect of Trial Type (*F*(1,18) = 1.33, *p* = 0.26, η^2^g = 0.002; congruent trials: *M* = 2.48, *SD* = 6.19; incongruent trials: *M* = 1.97, *SD* = 6.58) or interactions between Trial Type, Recording Area and Laterality on the amplitudes over the early time-window (200–500 ms; Trial Type and Recording Area: *F*(1,18) = 0.14, *p* = 0.71, η^2^g = 0.002; Trial Type and Laterality: *F*(2,36) = 0.83, *p* = 0.44, η^2^g = 0.002). When analyzing amplitudes in the late time-window (500–1000 ms), we found a main effect of Trial Type (*F*(1,18) = 5.17, *p* = 0.03, η^2^g = 0.01), with less positive amplitudes for incongruent (*M* = 1.31 μV; *SD* = 5.89 μV) than for congruent (*M* = 2.34 μV; *SD* = 5.22 μV) pairings (see Figure 2A–C). No interactions between Recording Area (*F*(1,18) = 0.11, *p* = 0.75, η^2^g = 0.001) or Laterality (*F*(2,36) = 1.07, *p* = 0.35, η^2^g = 0.005) were found. 

## 4. Discussion

A growing body of research has highlighted the presence of number-action associations in adults [43,75,76]. Here, we investigated brain activity by recording ERPs of 3- to 4-month-old infants while presented with both action and numerical information. We implemented a cueing paradigm [56,57] where infants were presented with two sequential images, the first image displaying a hand (closed or open) and the second a numerosity array (4 or 12 dots). These hand-number pairings were either congruent (i.e., small numerosity paired with a small hand opening) or incongruent (i.e., large numerosity paired with a small hand opening). When analyzing the data, we found different amplitudes over the time window of 500 to 1000 ms, suggesting that infants are capable of associating numerosities with hand actions, even at the age of three months.

Our findings extend previous results regarding the number-action link in infancy [55]. Specifically, our study supports the idea that in the first months of life humans spontaneously create expectations of congruency between magnitude representations associated with numerical and action-related information. The current study contributes neural evidence for the presence of this ability already in 3- to 4-month-old infants, and extends previous behavioral findings obtained in 8-month-old infants. In a previous behavioral study, only infants habituated to congruent number-hand pairings (but not to incongruent ones) looked longer at novel, incongruent test trials, suggesting that they are able to learn a congruency rule and apply it to novel instances [55]. Therefore, the present study adds an important contribution to the field: this is the first attempt to offer important insights for the knowledge and understanding of the neural basis of the number-action link, which, despite being behaviorally demonstrated in infancy, no previous studies have investigated it at the neural level. 

The present findings are also in line with theories that propose shared mechanisms for number and action. Recently, some authors have put forward the hypothesis of a *sensorimotor numerosity system* [14]. This system is thought to be involved in a variety of numerosity processes, not only in the perception of external stimuli but also in the production of numerosity-related actions. According to this view, the sensorimotor mechanism can be considered both a passive and an active system, which is dedicated to the perception of numerosity as well as to the planning and execution of self-produced actions. Moreover, due to the anatomical proximity of areas supporting both hand movement and numerosity representation, the neural basis of this system is presumably located in parietal areas [40,42]. 

Our study further supports the evidence of 3- to 4-month-old infants’ sensitivity for a correspondence between numerical and action-related stimuli, and an ability to integrate these two sources of information into a unified representation. Presumably, this unified representation allows infants to detect the congruency between the magnitude conveyed by a numerical quantity and a hand’s opening. Our results thus suggest the presence (and the activation) of the sensorimotor numerosity system already during the first months of life. The importance of having these types of processes connected at some processing level by a shared mechanism of magnitude is rather evident in daily activities. Starting from the first months, infants (and perhaps also newborns) need to consider related signals of magnitude present in their environment when planning and implementing actions by integrating these multiple sources of information. Therefore, the capacity to estimate the numerosity of the objects becomes fundamental to program an action directed to them and to execute it. To this scope, the integration of numerosity information and the magnitude of the action appears essential for a successful interaction with the environment.

Regarding the EEG signal, our brain response resembles that of the N400, the Nc and PSW components. Typically, the N400 has been found over the parietal and the Nc over the frontal recording sites, and they have been linked to meaning processing and attention engagement, respectively. The N400 component has been previously observed at the earliest 6 months of age in a lexical-semantic task [61], while the Nc has been documented soon after birth [77]. In our study, even though the incongruency effect was widely distributed over the scalp, the difference in amplitudes between the two trial types was more pronounced over the parietal than the frontal recording sites, suggesting that the component observed in our study resembles that of N400. Earlier linguistic studies in infants have shown that age and language skills contribute to the distribution of ERPs over the scalp. The ERPs are more widely distributed in younger than older infants [78]. Therefore, in our study, the incongruency effect observed over the frontal and parietal-occipital recordings can be seen, more likely, as a broader manifestation of the N400 component. Moreover, the cognitive process investigated by the present study is consistent with the effect demonstrated in previous studies. The presence of incongruent images leads to the violation of infants’ expectation, and this process can be comparable to the effects elicited by the unexpected events in the N400. 

The Nc has been often associated with allocation of attention and our response could be a manifestation of attentional engagement rather than conceptual understanding of relations. Infants may have engaged more attention for the incongruent trials compared to the congruent ones. However, if this was the case, this interpretation obligatorily implies that infants have previously created an abstract mapping between the pairings. 

Finally, another plausible component that could be linked to our results is the positive slow wave. This component has been already observed in association to unexpected outcomes over the frontal and the central areas [70]. In the study of Michel and colleagues, the PSW has been associated with a greater neural activity to process unexpected/unfamiliar stimuli and with a low-level process of elaboration. In line with this interpretation, our neural pattern can also be associated with an increased activity to elaborate incongruent stimuli and with a more rudimentary process. However, (and as in the case of Nc component), we believe that this processing can be considered a consequence of a conceptual mapping between magnitudes.

In conclusion, the findings from the present study pave the way for future research questions. One intriguing challenge is to understand the ontogenetic and/or evolutionary bases of the number–action link. Further work should therefore be aimed at establishing both the developmental and functional origins of the number–action link by proving whether this link is already functional at birth or emerges across development, and whether it is shared by other non-human animals.

## 5. Conclusions

The findings of the present study provide the first neural evidence for number and hand action association in the first months of life. Results showed that infants’ brain activity was modulated by the congruency/incongruency of number–action pairings over a distributed area (frontal and parietal-occipital). We thus suggest that this link can be found in adults and young children and is functional early in life, providing suggestions for future research. 

## Data Availability

Data are available at the following link: https://osf.io/jmvax/.
